# Retinal Protective Effect of Mono-Ethyl Fumarate in Experimental Age-Related Macular Degeneration via Anti-Oxidative and Anti-Apoptotic Alterations

**DOI:** 10.3390/ijms26041413

**Published:** 2025-02-07

**Authors:** Hara Lee, Siqi Zhang, Hong Ryul Ahn, Taejung Kim, Jiyool Kim, Heesu Lee, Sang Hoon Jung, Joonki Kim

**Affiliations:** 1 Center for Natural Product Efficacy Optimization, Natural Product Drug Development Division, Korea Institute of Science and Technology, Gangneung 25451, Republic of Korea; 122051@kist.re.kr (H.L.); 120060@kist.re.kr (S.Z.); hrahn@kist.re.kr (H.R.A.); kgsing@kist.re.kr (T.K.); kjy253@kist.re.kr (J.K.); 2 Natural Product Applied Science, KIST School, University of Science and Technology (UST), Gangneung 25451, Republic of Korea; 3 College of Dentistry, Gangneung Wonju National University, Gangneung 25457, Republic of Korea; nightsu@gwnu.ac.kr

**Keywords:** age-related macular degeneration (AMD), mono-ethyl fumarate, anti-oxidant, anti-apoptosis, ARPE-19, C57BL/6J, A2E, sodium iodate

## Abstract

Age-related macular degeneration (AMD) is a leading cause of vision impairment in people over the age of 60. Currently, the FDA-approved drugs for AMD have various side effects, and there is a notable lack of drug development for dry AMD. This study aimed to explore the therapeutic effects of mono-ethyl fumarate (MEF) on AMD. MEF effectively protected ARPE-19 cells from cell death induced by a combination of A2E and blue light exposure. In a C57BL/6J mouse model of retinal degeneration caused by sodium iodate, MEF played a role in preserving retinal thickness and maintaining the layered structure of the retina. It was assessed via fundus imaging, optical coherence tomography, and hematoxylin and eosin staining. Treatment with MEF significantly increased the expression of antioxidant proteins such as HO-1, NQO1, and SOD1 in ARPE-19 cells. Additionally, treatment with MEF significantly increased the levels of the antioxidant proteins SOD1 and GPX4 in the mouse retina. Concurrently, it significantly reduced the levels of apoptosis-related factors, such as the Bax/Bcl-2 ratio and Caspase -3 cleavage. These findings suggest that MEF may represent a promising therapeutic candidate for the management of AMD.

## 1. Introduction

Macular degeneration is a progressive and chronic condition that impacts the central region of the retina. It is commonly referred to as age-related macular degeneration (AMD) because it develops with advancing age [1]. AMD represents the primary cause of vision impairment among individuals aged 60 and older in developed countries. Furthermore, it is anticipated that the global prevalence of this condition will increase from 196 million cases in 2020 to an estimated 288 million by the year 2040 [2]. The advancement of AMD occurs in multiple stages (early, intermediate, advanced), each distinguished by the presence of drusen and alterations in pigmentation [3]. In early AMD, the majority of individuals remain asymptomatic, although the formation of drusen, which are yellow deposits located beneath the retina, may commence [4]. The progression from early to intermediate AMD is marked by an increase in the size of the drusen and the emergence of pigmentary alterations in the retina [4,5]. In intermediate AMD, the patient may demonstrate a decrease in reading speed, increased sensitivity to light, and difficulties adapting to changes in lighting conditions [6]. The advanced AMD can result in severe vision impairment and can be classified into two forms. One of these forms involves the development of atrophic regions, accompanied by progressive cell death of RPE and the loss of photoreceptors, known as geographic atrophy (GA) [7,8]. In order to receive a diagnosis of advanced GA, a patient must exhibit both atrophy and a significant decline in central vision. Peripheral visual acuity remains intact, even in the presence of the dry form of AMD [6]. Another form of advanced AMD is neovascular AMD, characterized by the proliferation of abnormal blood vessels in the macular area [9]. This can lead to the accumulation of subretinal and intraretinal fluid, bleeding, and fibrosis [10,11]. Neovascular AMD is associated with significant vision loss and progresses rapidly, making it a primary target for treatment [11,12]. Neovascular AMD is commonly referred to as wet AMD, while early, intermediate AMD, and GA are all classified as dry AMD [13].

In AMD, there is an impairment in the macular region, specifically the posterior segment of the central retina, which is critical for sharp and color vision [14]. The RPE, a single layer of cells situated between the choroid and the neural retina, plays a crucial role in preserving the health and functionality of photoreceptors [15]. A primary role of the RPE is to facilitate the transport of nutrients from the choroidal blood supply to the photoreceptors, which have high metabolic demands [16]. It also transports metabolic waste generated by photoreceptors to the bloodstream. RPE cells engage in the phagocytosis of the outer segments (OS) of photoreceptors that are shed daily, thereby recycling valuable components [17]. The melanin pigment present in RPE cells absorbs excess light, which helps prevent the disruption of visual images [18]. The RPE is able to form the outer blood–retinal barrier due to its polarized structure and tight cell-to-cell junctions. It serves as a selective barrier between the neural retina and the choroidal blood supply [19]. The pathological alterations observed in early AMD encompass the degeneration of RPE cells and modifications in cellular morphology. These changes contribute to the accumulation of metabolic byproducts, ultimately resulting in cellular apoptosis [20]. Based on the functions of RPE and its association with AMD, several studies have proposed that the RPE layer is a key area for the onset of the disease [21,22,23]. Therefore, most in vitro models focus on RPE cells, the fidelity of cellular systems to the in vivo biology of RPE, and their relevance to the pathogenesis of AMD [24].

Most of the Food and Drug Administration (FDA)-approved drugs for AMD to date aim to inhibit the activity of vascular endothelial growth factors (VEGFs). Such therapeutic interventions generally consist of the intravitreal injection of anti-VEGF agents, including bevacizumab and ranibizumab, aimed at mitigating neovascularization and edema, thereby stabilizing vision loss [12]. Nevertheless, these therapeutic interventions do not facilitate the recovery of vision that has already been irreversibly lost. A study indicated that one-third of patients with neovascular AMD experienced significant vision impairment after seven years of VEGF-A blockade therapy, and all patients exhibited signs of central retinal atrophy [25]. Additionally, repeated intravitreal injections over an extended period can lead to complications such as increased intraocular pressure, retinal detachment, uveitis, endophthalmitis, and subconjunctival hemorrhage [26]. On February 2023, the FDA approved SYFOVRE™ as the first treatment for GA, developed by Apellis Pharmaceuticals. The active compound of this medication is pegcetacoplan, which inhibits C3 [27]. Genetic variations in complementary C3, a key element of the three complement activation pathways, as well as the activation of C3 and the degradation of its active fragments, are significantly linked to an increased risk of developing both exudative and atrophic forms of AMD [28]. Pegcetacoplan binds to C3 and C3b, preventing the cleavage of C3 into C3a and C3b, as well as the production of downstream complement cascade mediators [29]. On August 2023, the FDA approved IZERVAY™ (avacincaptad pegol intravitreal solution) for the treatment of GA secondary to AMD. Avacincaptad pegol, a pegylated RNA aptamer, specifically binds to complement C5, inhibiting its cleavage and slowing the progression of complement-mediated inflammation and cellular death observed in GA [29]. However, the two recently approved drugs are also administered through intravitreal injection. Consequently, it is difficult to predict whether they will alleviate the existing complications associated with invasive treatment methods. There are no FDA-approved pharmacological treatments available for the early and intermediate stages of AMD, which affect 90% of all AMD patients. Therefore, individuals in these stages are only encouraged to maintain a healthy lifestyle, refrain from smoking, and consume antioxidants to help prevent vision loss [12]. Preventing significant vision impairment by attenuating the onset of AMD or delaying the progression of early and intermediate AMD to its late stages, such as GA or neovascular AMD, are effective strategies for managing this condition [30].

Numerous risk factors are associated with the onset of AMD. Among these, the systemic risk factors encompass hypertension, dyslipidemia, and diabetes mellitus [31,32,33]. The demographic and environmental risk factors encompass age, race, smoking, exposure to sunlight, and genetic predisposition [34]. Notably, oxidative stress plays a major role in age-related diseases, such as AMD, mainly due to the fact that the retina uses oxygen at a significantly higher rate compared to other tissues [35]. The increased production of reactive oxygen species (ROS) in the retina is affected by several factors. These include an intense oxygen metabolism, prolonged exposure to light, high concentrations of polyunsaturated fatty acids, and the presence of photosensitizers such as rhodopsin and lipofuscin. The excessive generation of ROS due to prolonged oxidative stress may exceed the antioxidant defenses of the retina. This can lead to alterations in and damage to carbohydrates, membrane lipids, proteins, and nucleic acids [36].

Mono-ethyl fumarate (MEF) is a component of fumarate (fumaric acid ester, FAE), an orally administered systemic medication used to treat psoriasis and multiple sclerosis. Licensed formulations of FAE include dimethyl fumarate (DMF) and three salts in MEF (calcium, zinc, and magnesium) [37]. DMF, the primary active ingredient in FAE, is known to oxidize the sulfhydryl group in Kelch-like ECH-associated protein 1. This subsequently results in the activation of nuclear factor erythroid 2-related factor 2 (Nrf2) [38]. Efforts are currently underway to apply this to various disease models [39,40]. In a study comparing the effects of DMF and MEF, it was found that MEF elevated Nrf2 protein levels in the nucleus via a different pathway to that of DMF. Additionally, MEF demonstrated a higher level of heme oxygenase 1(HO-1) mRNA than DMF at low concentrations [41]. However, despite the activation of antioxidant pathways and their clinical applications, MEF has not yet been thoroughly studied.

Lipofuscin serves as a significant source of ROS that accumulates within the RPE as a consequence of aging, resulting in heightened oxidative stress in the retina [42]. Lipofuscin predominantly comprises proteins that have undergone oxidative modification, as well as byproducts of lipolysis [43]. The composition comprises an intricate blend of pigments, with the primary hydrophobic constituent identified as N-retinylidene-N-retinylethanolamine (A2E) [44]. A2E is a bisretinoid compound that functions as a photosensitizer, producing superoxide anions when exposed to blue light at a wavelength of 430 nm. The photooxidized variant of A2E exhibits a high reactivity, which is likely implicated in the damage and subsequent apoptosis of RPE cells. Furthermore, it has been demonstrated to induce the damage of both DNA and proteins [45].

The retinal degeneration model induced by sodium iodate (SI) has been extensively studied, with studies looking at various doses, durations, and species. In murine models, the systemic delivery of SI has proven to be an effective method for inducing retinal degeneration characterized by the localized loss of RPE, thereby replicating many of the features associated with dry AMD [46,47,48]. These features include photoreceptor cell loss, RPE atrophy, glial membrane formation, and choriocapillaris degeneration, as seen in GA [49,50]. Hyperreflective foci formation and distortion of the RPE layer are some of the primary features of AMD [51]. The SI model serves as a valuable tool for understanding the clinical pathophysiology of this condition [52]. In mice injected with SI intraperitoneally, RPE cells initially began to undergo necrosis. Following this, degeneration of the photoreceptor segments adjacent to the necrotic RPE cells was observed [53]. In C57BL/6 mice, the administration of SI at a dosage of 10 mg/kg did not lead to retinal damage. Conversely, doses between 20 and 30 mg/kg of SI were associated with moderate levels of damage to the RPE and the retina. Furthermore, the administration of SI at doses exceeding 40 mg/kg led to severe retinal damage [46,47].

The objective of this study was to explore the therapeutic potential of MEF in AMD for the first time by assessing its protective effects on RPE cells and mouse retinas, building upon the antioxidant properties of MEF demonstrated in research on other diseases.

## 2. Results

### 2.1. The Protective Effects of MEF Against Damage Induced by A2E and Blue Light in ARPE-19 Cells

**Figure 1:**
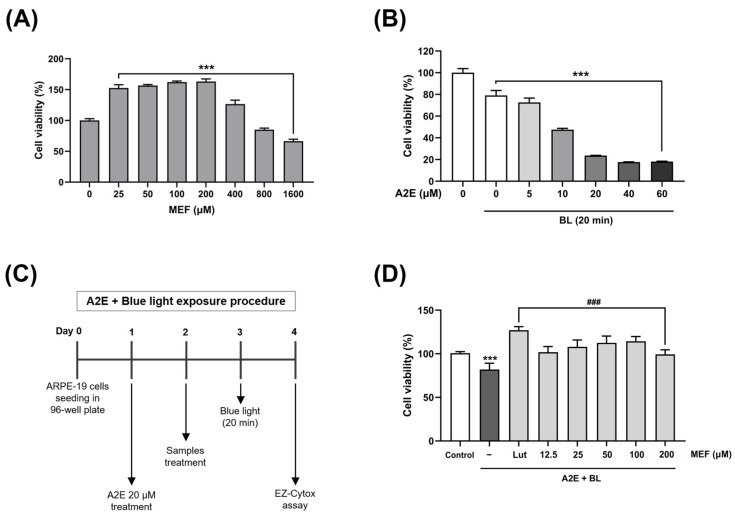
Treatment concentrations and cytoprotective effect of MEF against A2E and blue light-induced damage to ARPE-19 cells. (**A**) ARPE-19 cells were treated with various concentrations of MEF (25–1600 μM) and incubated for 48 h to measure cell viability. (**B**) ARPE-19 cells were treated with various concentrations of A2E (5–60 μM) and incubated for 24 h, followed by 20 min of blue light (480 nm, 6000 lux) illumination. Cell viability was measured 24 h after the illumination. (**C**) A schematic illustration of the sample treatment schedule. (**D**) ARPE-19 cells were treated with A2E (20 μM) for 24 h, after which the cells were treated with various concentrations of MEF (12.5–200 μM) and incubated for an additional 24 h. Then, the cells were illuminated with blue light (480 nm, 6000 lux) for 20 min and cell viability was measured 24 h post exposure to blue light. Treatment with 20 μM lutein was used as a positive control. All data are expressed as the mean ± standard deviation (SD). *** Indicates a significant difference (*p* < 0.001) from the untreated control group. ^###^ Indicates a significant difference (*p* < 0.001) from the A2E and blue light-exposed group. MEF, mono-ethyl fumarate; Lut, lutein; BL, blue light.

In order to assess the potential cytotoxic effects of MEF on ARPE-19 cells, we performed an evaluation of cell viability. After 48 h exposure to different concentrations of MEF (25–1600 μM), there was no cytotoxic effect observed in ARPE-19 cells treat with up to 400 μM MEF compared to the untreated control group (Figure 1A). To determine the optimal conditions for the combined treatment of A2E and blue light, ARPE-19 cells were exposed to various concentrations of A2E (5–60 μM), either alone or in combination with blue light. Treatment with A2E alone did not affect cell viability at concentrations up to 20 µM (Figure S1). However, when A2E-laden ARPE-19 cells were exposed to blue light, cell viability decreased in a concentration-dependent manner at all A2E concentrations (Figure 1B). The treatment with 20 µM of A2E, which led to the most significant differences based on the presence or absence of blue light, was selected as the condition for subsequent experiments. According to the experimental procedure involving treatment with A2E and blue light exposure (Figure 1C), various concentrations of MEF (12.5–200 μM) were administered to ARPE-19 cells. The MEF treatment was conducted in conjunction with A2E treatment and blue light exposure to assess the potential protective effects of MEF on cell viability. Exposure to A2E and blue light resulted in a significant reduction in ARPE-19 cell viability to 81.9 ± 7.2% in comparison to the untreated control group. The 20 μM lutein group, serving as a positive control, showed a significant increase in cell viability, measuring 127.1 ± 4.1%. All MEF treatment groups demonstrated significant improvements in cell viability, with values of 101.8 ± 6.5, 107.9 ± 7.8, 112.4 ± 7.9, 114.3 ± 5.6, and 99.4 ± 5.2% at various concentrations (12.5–200 μM) (Figure 1D).

### 2.2. The Effect of MEF on the Expression of Proteins Related to the Antioxidant Pathway in ARPE-19 Cells Exposed to A2E and Blue Light

**Figure 2:**
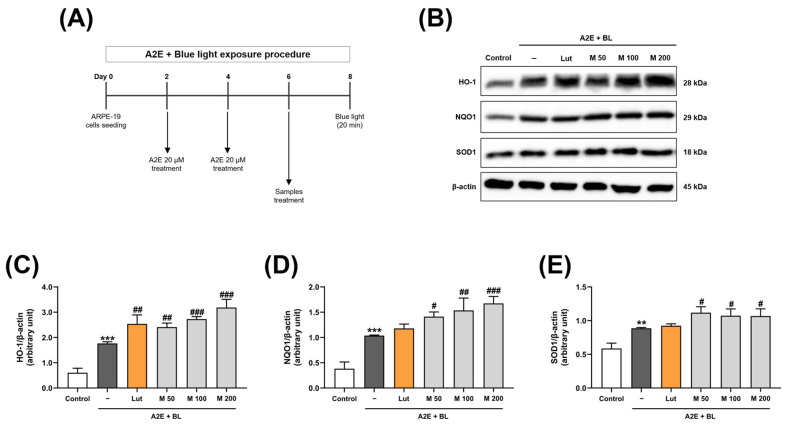
Analysis of antioxidant proteins in ARPE-19 cells exposed to A2E and blue light. (**A**) A schematic illustration of the treatment schedule for the sample treatment system. ARPE-19 cells were treated with 20 μM of A2E twice for 4 days, followed by treatments with various concentrations of MEF (50–200 μM) for 2 days. The cells were illuminated with blue light (480 nm, 6000 lux) for 20 min and then incubated for an additional 6 h. (**B**) Representative images of Western blot bands targeting HO-1, NQO1, SOD1, and β-actin proteins in ARPE-19 cells. (**C**–**E**) The expression levels of each protein were normalized to those of β-actin in ARPE-19 cells (N = 3). Treatment with 20 μM lutein was used as a positive control. All data are expressed as the mean ± SD. **, *** indicate significant differences (*p* < 0.01, 0.001) from the unexposed control group. ^#^, ^##^, ^###^ indicate significant differences (*p* < 0.05, 0.01, 0.001) from the A2E and blue light-exposed group. Lut, lutein; M, mono-ethyl fumarate; BL, blue light.

We evaluated the effects of MEF on the activation of antioxidant pathways in ARPE-19 cells. Following the experimental procedure involving A2E and blue light exposure (Figure 2A), we treated A2E-laden ARPE-19 cells with various concentrations of MEF (50–200 μM) and subsequently exposed them to blue light.

Six hours after illumination with blue light, the expression levels of HO-1, superoxide dismutase type 1 (SOD1), and NAD(P)H quinone dehydrogenase 1 (NQO1) proteins in ARPE-19 cell lysates were assessed using Western blot analysis (Figure 2B). HO-1 protein levels increased 2.9-fold compared to the unexposed control group as a result of A2E treatment and exposure to blue light. This change in HO-1 protein levels further rose to 4.2-fold in the unexposed control group undergoing treatment with 20 μM lutein. Furthermore, the MEF treatment at concentrations of 50, 100, and 200 μM resulted in a significant elevation in HO-1 protein levels in a concentration-dependent manner, with increases of 4.0-, 4.5-, and 5.3-fold, respectively, when compared to the unexposed control group (Figure 2C). Following exposure to A2E and blue light, there was a significant elevation in the levels of NQO1 protein, which exhibited a 2.7-fold increase in comparison to the unexposed control group. Following treatment with 20 μM lutein, the levels of NQO1 protein increased by 3.1-fold compared to the unexposed control group. Furthermore, treatment with MEF at concentrations of 50, 100, and 200 μM resulted in significant increases in NQO1 protein levels, with enhancements of 3.7-, 4.0-, and 4.4-fold, respectively, relative to the unexposed control group (Figure 2D). The levels of the SOD1 protein following A2E and blue light exposure were found to be elevated by 1.5-fold compared to the unexposed control group. The treatment with 20 μM lutein did not result in a statistically significant change in SOD1 protein levels. However, treatment with MEF at concentrations of 50, 100, and 200 μM led to a significant enhancement in SOD1 protein levels, with increases of 1.9-, 1.8-, and 1.8-fold, respectively, relative to the unexposed control group (Figure 2E).

### 2.3. The Protective Effect of MEF Against Pathological Changes and Reductions in Thickness in the Mouse Retina

**Figure 3:**
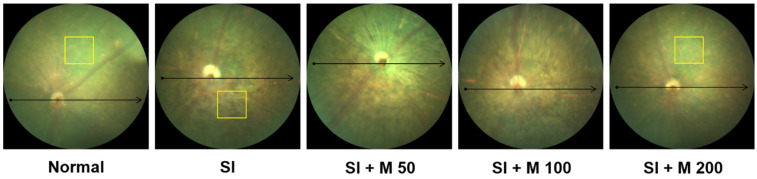
Representative fundus optical images of the eyes from mice with SI-induced retinal degeneration. Male C57BL/6J mice were orally administered various doses of MEF (50–200 mg/kg) once daily for 4 weeks. A single round of intraperitoneal injection of SI at a dose of 30 mg/kg was performed at the beginning of the second week. Pathological changes such as pigmentation of the central and peripheral retina were observed through fundus photography. The yellow box indicates the pigmented area of the retina. The horizontal axis of the arrow indicates the location on the retina where the OCT was captured. SI, sodium iodate; M, mono-ethyl fumarate.

**Figure 4:**
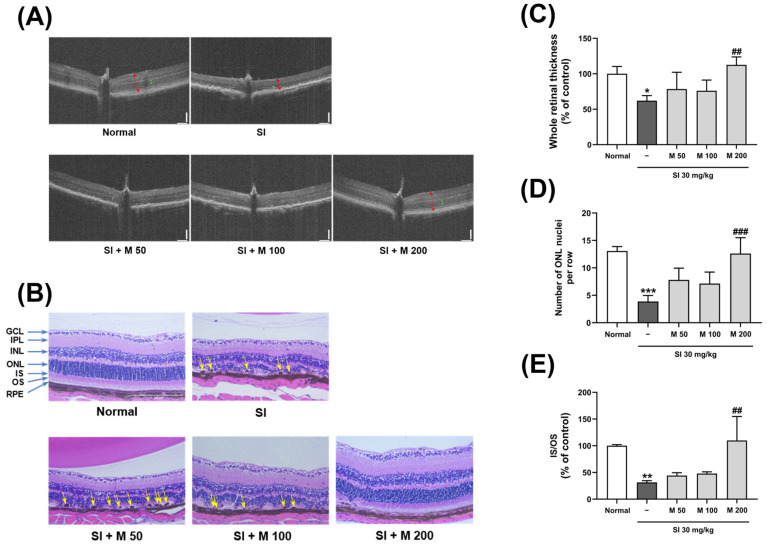
Assessment of retinal pathological changes using OCT imaging and H&E staining in mice with SI-induced retinal degeneration. Male C57BL/6J mice were orally administered with various doses of MEF (50–200 mg/kg) once daily for 4 weeks. A single round of intraperitoneal injection of SI at a dose of 30 mg/kg was performed at the beginning of the second week. (**A**) Representative OCT images of the central retinal region in experimental mice. Double-headed arrows mark total retinal thickness (red) and ONL thickness (green). (**B**) Representative retinal tissue sections stained with H&E (×200). Yellow arrowheads indicate the pigment cells that have migrated to the OS layer. Changes in the (**C**) whole retinal thickness, (**D**) number of ONL nuclei and (**E**) IS/OS thickness were quantified from the acquired H&E images (N = 3). All data are expressed as the mean ± SD. *, **, *** indicate significant differences (*p* < 0.05, 0.01, 0.001) from the normal group. ^##^, ^###^ indicate significant differences (*p* < 0.01, 0.001) from the SI-injected group. SI, sodium iodate; M, mono-ethyl fumarate, GCL, ganglion cell layer; IPL, inner plexiform layer; INL, inner nuclear layer; ONL, outer nuclear layer; IS, inner segment; OS, outer segment; RPE, retinal pigment epithelium.

To visualize the pathological changes and assess the effect of MEF on the mouse retina, non-invasive imaging and histological analyses were conducted (Figure 3 and Figure 4). The fundus optical images (Figure 3) revealed that the mice in the normal group exhibited characteristics of a healthy unaltered fundus. In comparison, mottling of the RPE was observed in mice that received SI injections. However, the administration of MEF at a dosage of 200 mg/kg provided protective effects against these changes.

Optical coherence tomography (OCT) images were obtained to examine the structure and thickness of the retina (Figure 4A) and no indications of retinal damage were detected in the normal mice. Conversely, the retinas of mice that received SI injections exhibited a loss of lamination, characterized by a decrease in overall retinal thickness and a significant reduction in the ONL. These changes, which indicate damage to the retina, were prevented by administering MEF at a dose of 200 mg/kg. The results obtained from fundus optical images and OCT images were validated through a histological examination of retinal tissues stained with hematoxylin and eosin (H&E) (Figure 4B). Following SI injection, the whole retinal thickness of the mice was significantly reduced by 62.0 ± 7.3% compared to the normal group. This decrease was observed concurrently with the migration of pigmented cells into the OS layer. The administration of MEF at a dosage of 200 mg/kg resulted in a retinal thickness measuring 112.5 ± 11.3% of that observed in the normal group (Figure 4C), providing significant protection against the thinning of whole retinal thickness. This protective effect was accompanied by a reduction in the migration of pigmented cells. After measuring the number of ONL nuclei per line (Figure 4D), a significant reduction to 29.6% was observed in the SI-injected group compared to the normal group. However, the administration of 200 mg/kg MEF significantly preserved the number of ONL nuclei per row, maintaining it at 96.4% of the normal group. Consequently, we measured the thickness of the inner segment (IS) and OS layers of photoreceptors (Figure 4E). The injection of SI resulted in a significant reduction in IS/OS thickness to 31.4% compared to the normal group. Conversely, treatment with 200 mg/kg of MEF preserved the IS/OS thickness at 109.9% of the normal group, indicating a significant protective effect on the retinal structure of the mice.

### 2.4. The Effect of MEF on the Expression of Proteins Related to the Antioxidant Pathway in the Retinas of Mice with Retinal Degeneration Induced by SI

**Figure 5:**
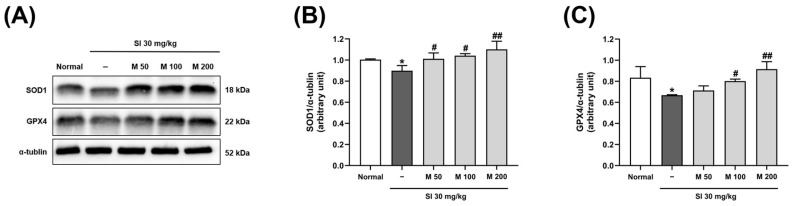
Analysis of antioxidant proteins expression in the retina of SI-injected mice. Male C57BL/6J mice were orally administered with various doses of MEF (50–200 mg/kg) once daily for 4 weeks. A single round of intraperitoneal injection of SI at a dose of 30 mg/kg was performed at the beginning of the second week. (**A**) Representative images of Western blot bands targeting SOD1, GPX-4, and α-tubulin proteins from three independent experimental mice (N = 3). (**B**,**C**) The expression levels of each protein were normalized to those of α-tubulin from the experimental mice. All data are expressed as the mean ± SD. * indicates a significant difference (*p* < 0.05) from the normal group. ^#^, ^##^ indicate significant differences (*p* < 0.05, 0.01) from the SI-injected group. SI, sodium iodate; M, mono-ethyl fumarate.

To assess the effects of MEF on antioxidant protein levels, we evaluated the expression of SOD1 and glutathione peroxidase 4 (GPX4) proteins in retinal tissues using Western blot analysis (Figure 5A). Following the injection of the SI, the protein levels of SOD1 showed a significant reduction, reaching only 89.5% of those observed in the normal group. However, the administration of MEF at doses of 50, 100, and 200 mg/kg resulted in a significant restoration of SOD1 protein levels in a dose-dependent manner, achieving levels of 100.8%, 103.7%, and 109.8%, respectively (Figure 5B). The protein levels of GPX4, another antioxidant protein, was reduced to 80.1% of the normal group following SI injection. The GPX4 protein levels were significantly restored to 96.2% and 109.8% of the normal group following the administration of 100 and 200 mg/kg of MEF, respectively (Figure 5C).

### 2.5. The Effect of MEF on the Expression of Apoptosis Pathway-Related Proteins in the Retinas of Mice with SI-Induced Retinal Degeneration

**Figure 6:**
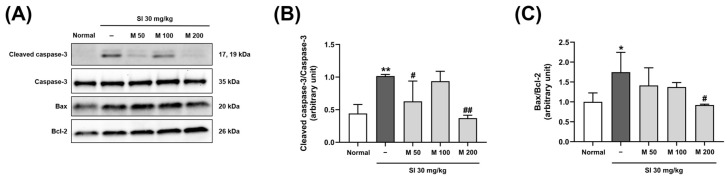
Analysis of apoptosis-related proteins expression in the retina of SI-injected mice. Male C57BL/6J mice were orally administered with various doses of MEF (50–200 mg/kg) once daily for 4 weeks. A single round of intraperitoneal injection of SI at a dose of 30 mg/kg was performed at the beginning of the second week. (**A**) Representative images of Western blot bands targeting Caspase-3, Bax, Bcl-2, and α-tubulin proteins from three independent experimental mice (N = 3). (**B**,**C**) The expression levels of each protein were normalized to those of α-tubulin or the total form of the target protein in experimental mice. All data are expressed as the mean ± SD. *, ** indicate significant differences (*p* < 0.05, 0.01) from the normal group. ^#^, ^##^ indicate significant differences (*p* < 0.05, 0.01) from the SI-injected group. SI, sodium iodate; M, mono-ethyl fumarate.

A Western blot analysis was performed to quantify the levels of apoptosis-related proteins, specifically Bcl2-associated X (Bax), B-cell lymphoma-2 (Bcl-2), and caspase-3 (Figure 6A). This analysis aimed to investigate the effects of MEF administration at dosages of 50, 100, and 200 mg/kg on the apoptosis pathway in the retinas of mice injected with SI. We evaluated the Bax to Bcl-2 ratio, which was used as an indicator of cell death susceptibility (Figure 6B). Following SI injection, the Bax/Bcl-2 ratio significantly increased to 1.7-fold that of the normal group. The administration of MEF at a dosage of 200 mg/kg reduced this increase; the ratio was significantly decreased to 0.9-fold that of the control group. The level of caspase-3 activation was assessed by calculating the ratio of cleaved caspase-3 to the total amount of caspase-3 (Figure 6C). This ratio significantly increased to 2.3-fold that of the normal group after SI injection, but after the administration of 200 mg/kg of MEF, it significantly decreased to 0.8-fold compared to that of the normal group.

## 3. Discussion

The human retina has a yellowish region known as the macula, which measures about 5 to 6 mm across. The macula is densely populated with cone photoreceptors, which are essential for high-acuity vision [54]. The macula, which has high metabolic demands, not only exhibits elevated physiological levels of ROS for signal transduction but also generates significant amounts of ROS during cellular metabolism [55]. Although the retina and RPE cells are rich in both enzymatic and non-enzymatic antioxidants, ROS levels increase in aged retinas [56]. Excessive oxidative stress, caused by increased levels of ROS and a weakened antioxidant cellular defense system, leads to changes and damage to carbohydrates, membrane lipids, proteins, and nucleic acids. This can result in damage to photoreceptors, RPE cells, and the choroid during the process of apoptosis [36,56]. The RPE is composed of a single layer of cells located on Bruch’s membrane, which perform various functions to maintain the homeostasis of the ocular system. They support visual function and uphold ocular immunity by establishing the blood–retinal barrier, protecting the retina from oxidative stress through light absorption, transporting nutrients, and removing waste products generated by the photoreceptor metabolism. Because of these critical functions, any dysfunction in the RPE can lead to diseases that affect human vision, such as AMD [57].

A2E is a compound that accumulates in the RPE as individuals age. It is known to generate superoxide anions and cause damage to RPE cells through DNA and protein denaturation when oxidized by blue light [45]. In this study, we utilized an experimental model consisting of ARPE-19 cells, a cell line derived from human RPE. ARPE-19 cells were exposed to a treatment involving the use of both A2E and blue light to induce damage. It has been reported that this treatment exposes cells to an oxidative stress environment through excessive ROS generation [58,59].

The primary line of antioxidant defense in cells is provided by antioxidant enzymes, including SOD, catalase, and GPX [60]. When cells are exposed to low doses of oxidants, their resistance to high doses of oxidants can be enhanced through an increase in antioxidant enzymes [61,62]. The second line of antioxidant defense is represented by exogenous small molecule antioxidants derived from the diet, such as vitamins C and E, carotenoids, and flavonoids [60]. However, if these antioxidant defense and repair mechanisms are insufficient to protect cells, apoptosis may be activated as a third protective mechanism to directly eliminate oxidized biomolecules [60,63]. When A2E-laden ARPE-19 cells were exposed to blue light, an increase in antioxidant enzymes, including SOD, HO-1, and NQO1 was observed 6 h post illumination. This is suggested to be a function of the cell’s primary antioxidant defense in environments with low oxidative stress. Treatment with MEF further elevated the levels of these antioxidant enzymes, suggesting that MEF functioned as a secondary line of antioxidant defense, thereby enhancing the overall antioxidant defenses in the cells. The HO-1 protein expression changes observed in ARPE-19 cells exposed to A2E and blue light are consistent with previous reports stating that the nuclear translocation of Nrf2 and increased cytoplasmic levels of the HO-1 protein are induced by oxidative stress. However, conflicting results were shown regarding SOD levels in ARPE-19 cells exposed to A2E and blue light. These discrepancies may be attributed to differences in the experimental method or the measurement of total SOD versus specific types of SOD [64]. Significant cell death was observed 24 h after exposure to blue light. This cell death be triggered by excessive oxidative stress within the cells. MEF significantly protected ARPE-19 cells from cell death by enhancing antioxidant activity in cells with disrupted redox homeostasis.

A single dose of SI via intraperitoneal injection into C57BL/6J mice results in significant RPE damage, accompanied by visual impairment, dysfunction, and photoreceptor loss [48]. These alterations resemble GA, a progressive form of dry AMD characterized by the irreversible loss of RPE cells, along with the degeneration of the adjacent neuroretina and choroid [50]. Previous studies have demonstrated that the injection of SI into C57BL/6J mice resulted in a significant reduction in both IS/OS thickness and overall retinal thickness, with a particularly significant decrease in ONL thickness, which contains photoreceptor nuclei [46]. Changes in the mouse retina resulting from the injection of SI and MEF were confirmed through fundus optical images and OCT imaging. A further examination of specific retinal layers was conducted using a histological analysis with H&E staining. As a result, mice injected with SI exhibited uneven pigmentation in the retina and a reduction in the thickness of the whole retinal layer. A particularly significant reduction in the ONL, characteristic of SI-induced retinal degeneration [48,65], was observed along with the migration of pigmented cells to the OS layer. The results indicate that SI-induced retinal degeneration was effectively elicited. The administration of MEF prevented the mottling of the RPE, inhibited the migration of pigment cells to the OS layer, and mitigated the reduction in whole retinal thickness induced by SI. The death of RPE cells, followed by the degeneration of photoreceptor cells, is considered a hallmark of AMD [20]. Specifically, MEF effectively protected the mouse retina from the adverse changes induced by SI by preventing the thinning of both the ONL and IS/OS layer. In mouse models of SI-induced retinal degeneration, increased oxidative stress and the resulting retinal damage are well documented [46,66,67,68]. To further elucidate the pathological results, protein levels in the mouse retina were assessed using Western blot analysis. Increased levels of cleaved caspase-3 protein, confirmed through immunohistochemical staining, were reported in SI-induced retinal degeneration mice [69]. The balance between the anti-apoptotic protein Bcl-2 and the pro-apoptotic protein Bax is known to regulate apoptosis by influencing the activity of downstream apoptotic proteins, such as caspase-3 and caspase-9 [70]. The cleavage of Poly (ADP-ribose) polymerase 1 (PARP1) leads to a loss of DNA repair activity, and the 89 kDa PARP1 fragment may promote apoptosis by inducing the release of the apoptosis-inducing factor from the mitochondria [71]. A downregulation of antioxidant proteins, including SOD-1 and GPX4, was observed following the injection of SI. It is assumed that this phenomenon results from the breakdown of the antioxidant defense system, which occurs when oxidative stress significantly exceeds the antioxidant capacity of retinal tissue. Consistent with this expectation, the apoptotic pathway was activated through the increased Bax/Bcl-2 ratio and the cleavage of caspase-3 and PARP-1 (Figure S2) in the retinas receiving SI injections. The administration of MEF reversed all of these negative changes. These results suggest that MEF effectively protected the mouse retina against retinal damage caused by SI by suppressing the apoptosis pathway through the upregulation of antioxidant enzymes.

Future investigations are essential to uncover the full details of the therapeutic mechanisms through which MEF exerts its protective effects against AMD. Specifically, the utilization of electroretinography to evaluate photoreceptor function is anticipated to further support the potential of MEF as a treatment candidate for AMD. Furthermore, evaluating the long-term efficacy and safety of MEF in preclinical and clinical studies will be crucial for determining its viability as a treatment for dry AMD. The results of this study suggest that MEF holds promise as a therapeutic candidate, not only for preventing AMD but also for potentially slowing the progression of existing dry AMD, making it an exciting avenue for future research and development.

## 4. Materials and Methods

### 4.1. ARPE-19 Cell Line

Human RPE cells (ARPE-19, #CRL-2302) were obtained from the American Type Culture Collection (Manassas, VA, USA). The cells were maintained in Dulbecco’s modified Eagle’s medium F-12 (Cytiva, Marlborough, MA, USA, #SH30023.01), supplemented with 10% fetal bovine serum (Thermo Fisher Scientific, Waltham, MA, USA) and 1% Anti-Anti (Thermo Fisher Scientific, USA) at 37 °C under 5% CO_2_. ARPE-19 cells were passaged twice a week using TrypLE (Thermo Fisher Scientific, USA) when confluency was reached in 90% of the plates.

### 4.2. Evaluation of Cell Viability

ARPE-19 cells were seeded in 96-well plates at a concentration of 1.0 × 10^4^ cells per well and grown for 24 h. ARPE-19 cells were exposed to various concentrations of MEF (25–1600 μM) for 48 h or A2E (5–60 μM) for 24 h. Cell viability was measured using an EZ–cytox assay kit (DoGenBio, Seoul, Republic of Korea, #EZ-3000). Briefly, cells were treated with an EZ–cytox solution and incubated for 1.5 h. Subsequently, cell viability was assessed through measuring absorbance at 450 nm using a multi-mode microplate reader (Varioskan LUX; Thermo Fisher Scientific, USA).

To assess blue light-induced cytotoxicity in A2E-laden ARPE-19 cells, the cells were treated with various concentrations of A2E (5–60 μM) for 24 h. Each well was then washed twice with phosphate-buffered saline (PBS) and filled with PBS. The cells were exposed to blue light (480 nm, 6000 lux) for 20 min using a specially designed blue light illumination device. Following light exposure, the PBS was replaced with the culture medium, and the cells were incubated for another 24 h. Cell viability was measured using EZ–cytox assay.

### 4.3. A2E with Blue Light Exposure Procedure

ARPE-19 cells were seeded into 96-well plates at a density of 1.0 × 10^4^ cells per well and grown for 24 h. ARPE-19 cells were treated with 20 μM A2E and incubated for 24 h. The cultured medium was removed and the wells were washed with PBS. Various concentrations of MEF (12.5–200 μM) or 20 μM of lutein were used for treatment and the cells were maintained for 24 h. After washing the wells twice with PBS, the wells were filled with PBS and exposed to blue light (480 nm, 6000 lux) for 20 min. PBS was replaced with medium, and the cells were maintained for 24 h. Cell viability was assessed via EZ–cytox assay.

To obtain cell lysate, ARPE-19 cells were seeded at 70% confluency in a culture dish. Subsequently, they were treated with 20 μM A2E twice (on days 2 and 4) over a period of 4 days. On day 6, the cultured medium was removed and the dishes were washed with PBS. The cells were then treated with MEF (50–200 μM) or with 20 μM lutein for 2 days. Following this treatment, the dishes were washed twice with PBS and filled with PBS. Subsequently, ARPE-19 cells were subjected to blue light exposure (480 nm, 6000 lux) for 20 min and then incubated for an additional 6 h.

All experimental methods using A2E and blue light were redesigned based on a protocol described previously by Kim et al. (2020) [64].

### 4.4. Animals

Six-week-old C57BL/6J mice were obtained from Orient Bio (Seongnam, Republic of Korea). All animals were housed for one week in a facility with free access to normal food and water, maintained at a temperature of 23–25 °C and humidity of 40–50%, with a 12 h light/dark cycle. The study design adhered to the ethical guidelines and all procedures involving animals complied with the principles of good animal experimental practice. Approval for the study protocol was granted by the Research Ethics Committee of the Korea Institute of Science and Technology (Approval Code: KIST-5088-2022-06-091).

### 4.5. Retinal Degeneration Mouse Model Induced by SI Injection

After a week of acclimatization, the mice were randomly assigned to five groups (N = 9 for each group):

- 1. Normal: vehicle.
- 2. SI: vehicle + SI 30 mg/kg.
- 3. M 50: MEF 50 mg/kg + SI 30 mg/kg.
- 4. M 100: MEF 100 mg/kg + SI 30 mg/kg.
- 5. M 200: MEF 200 mg/kg + SI 30 mg/kg.

**Figure 7:**
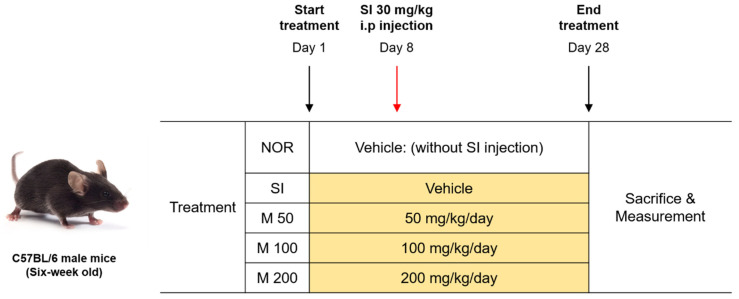
The experimental groups and animal treatment schedule for the induction of retinal degeneration via SI injection. Male C57BL/6J mice in the normal and SI groups were orally administered with 0.45% β-cyclodextrin as the vehicle for a duration of 4 weeks. Mice in the MEF groups were orally administered with 50, 100, and 200 mg/kg of MEF. A single round of an intraperitoneal injection of SI at a dosage of 30 mg/kg was injected at the beginning of the second week to all mice, except those in the normal group. One day before the end of the 4-week administration schedule, fundus optical images and OCT images of the mice were obtained. On the final day of administration, the mice were sacrificed, and their eyes were collected for tissue staining and Western blot analysis.

The treatment protocol for the mice was outlined previously (see Figure 7). Briefly, mice were orally administered with the vehicle or various doses of MEF (50, 100, 200 mg/kg) for a duration of 4 weeks. At the beginning of the second week, all mice, except those in the normal group, received a single injection of SI at a dose of 30 mg/kg via intraperitoneal injection. MEF was dissolved in a 0.45% β-cyclodextrin solution and administered orally. One day before the end of the 4-week administration schedule, fundus optical images and OCT images of the mice were obtained. On day 28, the mice were euthanized through the inhalation of a lethal dose of the anesthetic isoflurane. Subsequently, the eyes were collected for histological analysis and the quantification of protein levels. The retinas were isolated from the eyes for subsequent Western blot analysis. During the whole experiment, no experimental animals showed any signs of drug toxicity, including significant weight changes or behavioral abnormalities.

### 4.6. Fundus and OCT

OCT images and fundus optical images were obtained one day prior to sacrifice. The mice were anesthetized via an intraperitoneal injection of a combination of the veterinary anesthetics Zoletil^®^ 50 (Virbac, Carros, France) and Rompun^®^ inj. (Bayer, Leverkusen, Germany). Mydrin^®^-P (Santen Pharmaceutical Co., Ltd., Shiga, Japan) was instilled onto the ocular surface to dilate the pupils. The mice were instilled with the ophthalmic lubricant hycell solution 2% (Samil-pharm, Seoul, Republic of Korea) to protect their eyes from dryness and physical damage from the imaging device. OCT images and fundus optical images were captured using Micron Reveal software (Phoenix-Micron, Bend, OR, USA, https://www.micron.com/) in conjunction with the MICRON^®^ IV Retinal imaging Microscope (Phoenix-Micron, Bend, OR, USA).

### 4.7. Histological Analysis (Hematoxylin and Eosin Staining)

Retinal tissues fixed in 10% formalin were embedded in paraffin and sectioned into 4 μm thick slices. H&E images were obtained using a Leica DM IL microscope (Leica Camera AG; Wetzlar, Germany) in conjunction with a PL-A622C camera (Pixelink; Ottawa, ON, Canada). The thicknesses of the whole retina, ONL, and IS/OS were measured using ImageJ software version 1.53t (US National Institutes of Health, Bethesda, MD, USA). From each mouse’s retina, five locations starting from either side of the optic nerve were assessed for H&E analysis. The five measurements were averaged to obtain the mean thicknesses of whole retina, ONL, and IS/OS. Three mice were analyzed from each experimental group (N = 3).

### 4.8. Western Blotting

ARPE-19 cells and retinal tissues were harvested and lysed using RIPA Lysis and Extraction Buffer (Thermo Fisher Scientific, USA, #89900), which contained 1× Halt™ Protease and Phosphatase Inhibitor Cocktail (Thermo Fisher Scientific, USA, #748441). Lysates were incubated on ice for 1 h with vortexing every 10 min and centrifuged at 20,000× g for 20 min at 4 °C, and the supernatant was collected in a new tube. Protein concentration was measured using the Bradford assay. Proteins weighing 15–20 μg were separated via 10% sodium dodecyl sulfate–polyacrylamide gel electrophoresis and then transferred to a 0.2 μm poly-vinylidene difluoride membrane. The membrane was blocked using EveryBlot Blocking buffer (Bio-Rad, Hercules, CA, USA, #12010020) and then incubated with primary antibodies (1:1000) overnight at 4 °C. The membrane was washed with TBST and then incubated with HRP-conjugated secondary antibodies (1:2000) for 1 h at room temperature. These immunoreactive membranes were visualized using the ECL Substrate (Bio-Rad, USA, #170-5060) or Femto Substrate (Thermo Fisher, USA, #34095). Imaging and densitometry of these bands were performed using iBright CL1000 Imaging System (Thermo Fisher, USA). Protein band intensities were measured using ImageJ software and normalized to β-actin or α-tubulin.

### 4.9. Antibodies and Chemicals

The following antibodies were purchased from Cell Signaling Technology, Inc. (Danvers, MA, USA): SOD1 (#37385), Cleaved Caspase-3 (#9664), Caspase-3 (#9662), Bax (#2772), Bcl-2 (#3498), β-actin (#4970), α-tubulin (#3873), and cleaved PARP (#5625). The following antibodies were obtained from Proteintech (Rosemont, IL, USA): NQO1 (#11451-1-AP), HO-1/HMOX1 (#10701-1-AP), and PARP1 (#22999-1-AP). Additionally, the GPX4 (#ab125066) antibody was obtained from Abcam (Cambridge, UK).

All chemicals were obtained from Sigma Aldrich (St. Louis, MO, USA). A2E was supplied by Dr. Taejung Kim from the Korea Institute of Science and Technology. Briefly, A2E was synthesized by dissolving 100 mg of all-trans-retinal and 9.5 mg of ethanolamine in 3 mL of ethanol, to which 4.5 μL of acetic acid was added before stirring in the dark for 3 days. The conversion of all-trans-retinal to iminium was confirmed via UPLC/MS. When the ratio of iminium no longer changed, acetic acid (160 μL, 2.80 mmol) was added at room temperature and stirred in the dark for 2 days. After the mixture was concentrated in vacuo, the residue was purified via HPLC [Phenomenex Luna^®^ 5 μm C18(2) 100 Å LC Column 250 × 10 mm, 80–96% ACN/water (0.1% TFA) for 60 min, 2.0 mL/min flow detected at UV 339 nm] [72]. A2E and *iso-*A2E were detected at retention times (*t*_R_) of 16.7 min and 18.8 min, respectively. The collection of each fraction provided pure A2E and *iso-*A2E for further analysis. The purified A2E was diluted to 10 mM in dimethyl sulfoxide and stored at −20 °C until use.

### 4.10. Statistical Analysis

Statistical analyses were performed using a one-way ANOVA, followed by Holm-Šídák’s tests utilizing Prism (10.3 version; GraphPad Software, Inc., San Diego, CA, USA). The results are presented as mean ± SD (N ≥ 3) and a *p*-value of less than 0.05 was considered statistically significant in all cases.

## 5. Conclusions

In summary, this study was conducted to explore the potential of MEF as a therapeutic agent for AMD. The MEF treatment significantly protected ARPE-19 cells damaged by A2E and blue light exposure from cell death by increasing the level of antioxidant proteins. In addition, MEF significantly protected the mouse retina from SI-induced retinal degeneration in a mouse model by increasing the level of antioxidant proteins and regulating apoptosis-related factors. Suggesting the need for additional future investigations on the detailed therapeutic mechanisms against AMD, our current results suggest the potential of MEF as a therapeutic candidate for preventing and treating dry AMD.

## Data Availability

All data are available from the corresponding author upon reasonable request.

## Supplementary Materials

The following supporting information can be downloaded at: https://www.mdpi.com/article/10.3390/ijms26041413/s1.

## Abbreviations

A2E	N-retinylidene–N-retinylethanolamine
AMD	Age-related macular degeneration
Bax	Bcl2-associated X
Bcl-2	B-cell lymphoma-2
DMF	Dimethyl fumarate
FAE	Fumaric acid ester
FDA	Food and Drug Administration
GA	Geographic atrophy
GPX4	Glutathione peroxidase 4
H&E	Hematoxylin and eosin
HO-1	Heme oxygenase 1
IS	Inner segment
MEF	Mono-ethyl fumarate
NQO1	NAD(P)H Quinone Dehydrogenase 1
Nrf2	Nuclear factor erythroid 2-related factor 2
OCT	Optical coherence tomography
ONL	Outer nuclear layer
OS	Outer segment
PARP1	Poly (ADP-ribose) polymerase 1
PBS	Phosphate-buffered saline
ROS	Reactive oxygen species
RPE	Retinal pigment epithelium
SI	Sodium iodate
SOD1	Superoxide dismutase type 1
VEGF	Vascular endothelial growth factor

## References

[1] Deng Y., Qiao L., Du M., Qu C., Wan L., Li J., Huang L. (2022). Age-related macular degeneration: Epidemiology, genetics, pathophysiology, diagnosis, and targeted therapy. Genes. Dis..

[2] Wong W.L., Su X., Li X., Cheung C.M., Klein R., Cheng C.Y., Wong T.Y. (2014). Global prevalence of age-related macular degeneration and disease burden projection for 2020 and 2040: A systematic review and meta-analysis. Lancet Glob. Health.

[3] Miller J.W., Bagheri S., Vavvas D.G. (2017). Advances in Age-related Macular Degeneration Understanding and Therapy. US Ophthalmic Rev..

[4] Gehrs K.M., Anderson D.H., Johnson L.V., Hageman G.S. (2006). Age-related macular degeneration--emerging pathogenetic and therapeutic concepts. Ann. Med..

[5] Flores R., Carneiro Â., Tenreiro S., Seabra M.C. (2021). Retinal Progression Biomarkers of Early and Intermediate Age-Related Macular Degeneration. Life.

[6] Vyawahare H., Shinde P. (2022). Age-Related Macular Degeneration: Epidemiology, Pathophysiology, Diagnosis, and Treatment. Cureus.

[7] Pfau M., von der Emde L., de Sisternes L., Hallak J.A., Leng T., Schmitz-Valckenberg S., Holz F.G., Fleckenstein M., Rubin D.L. (2020). Progression of Photoreceptor Degeneration in Geographic Atrophy Secondary to Age-related Macular Degeneration. JAMA Ophthalmol..

[8] Danis R.P., Lavine J.A., Domalpally A. (2015). Geographic atrophy in patients with advanced dry age-related macular degeneration: Current challenges and future prospects. Clin. Ophthalmol..

[9] Michalska-Małecka K., Kabiesz A., Nowak M., Śpiewak D. (2015). Age related macular degeneration–challenge for future: Pathogenesis and new perspectives for the treatment. Eur. Geriatr. Med..

[10] Fleckenstein M., Schmitz-Valckenberg S., Chakravarthy U. (2024). Age-Related Macular Degeneration: A Review. JAMA.

[11] Flores R., Carneiro Â., Vieira M., Tenreiro S., Seabra M.C. (2021). Age-Related Macular Degeneration: Pathophysiology, Management, and Future Perspectives. Ophthalmologica.

[12] Thomas C.N., Sim D.A., Lee W.H., Alfahad N., Dick A.D., Denniston A.K., Hill L.J. (2022). Emerging therapies and their delivery for treating age-related macular degeneration. Br. J. Pharmacol..

[13] El-Den N.N., Naglah A., Elsharkawy M., Ghazal M., Alghamdi N.S., Sandhu H., Mahdi H., El-Baz A. (2023). Scale-adaptive model for detection and grading of age-related macular degeneration from color retinal fundus images. Sci. Rep..

[14] Fleckenstein M., Keenan T.D.L., Guymer R.H., Chakravarthy U., Schmitz-Valckenberg S., Klaver C.C., Wong W.T., Chew E.Y. (2021). Age-related macular degeneration. Nat. Rev. Dis. Primers.

[15] Yang S., Zhou J., Li D. (2021). Functions and Diseases of the Retinal Pigment Epithelium. Front. Pharmacol..

[16] Chao J.R., Knight K., Engel A.L., Jankowski C., Wang Y., Manson M.A., Gu H., Djukovic D., Raftery D., Hurley J.B. (2017). Human retinal pigment epithelial cells prefer proline as a nutrient and transport metabolic intermediates to the retinal side. J. Biol. Chem..

[17] Kim J.Y., Zhao H., Martinez J., Doggett T.A., Kolesnikov A.V., Tang P.H., Ablonczy Z., Chan C.C., Zhou Z., Green D.R. (2013). Noncanonical autophagy promotes the visual cycle. Cell.

[18] Lakkaraju A., Umapathy A., Tan L.X., Daniele L., Philp N.J., Boesze-Battaglia K., Williams D.S. (2020). The cell biology of the retinal pigment epithelium. Prog. Retin. Eye Res..

[19] Naylor A., Hopkins A., Hudson N., Campbell M. (2019). Tight Junctions of the Outer Blood Retina Barrier. Int. J. Mol. Sci..

[20] Si Z., Zheng Y., Zhao J. (2023). The Role of Retinal Pigment Epithelial Cells in Age-Related Macular Degeneration: Phagocytosis and Autophagy. Biomolecules.

[21] Somasundaran S., Constable I.J., Mellough C.B., Carvalho L.S. (2020). Retinal pigment epithelium and age-related macular degeneration: A review of major disease mechanisms. Clin. Exp. Ophthalmol..

[22] Wang S., Li W., Chen M., Cao Y., Lu W., Li X. (2023). The retinal pigment epithelium: Functions and roles in ocular diseases. Fundam. Res..

[23] Bird A.C. (2010). Therapeutic targets in age-related macular disease. J. Clin. Investig..

[24] Bharti K., den Hollander A.I., Lakkaraju A., Sinha D., Williams D.S., Finnemann S.C., Bowes-Rickman C., Malek G., D’Amore P.A. (2022). Cell culture models to study retinal pigment epithelium-related pathogenesis in age-related macular degeneration. Exp. Eye Res..

[25] Rofagha S., Bhisitkul R.B., Boyer D.S., Sadda S.R., Zhang K. (2013). Seven-year outcomes in ranibizumab-treated patients in ANCHOR, MARINA, and HORIZON: A multicenter cohort study (SEVEN-UP). Ophthalmology.

[26] Falavarjani K.G., Nguyen Q.D. (2013). Adverse events and complications associated with intravitreal injection of anti-VEGF agents: A review of literature. Eye.

[27] FDA Approves SYFOVRE™ (Pegcetacoplan Injection) as the First and Only Treatment for Geographic Atrophy (GA), a Leading Cause of Blindness. https://investors.apellis.com/news-releases/news-release-details/fda-approves-syfovretm-pegcetacoplan-injection-first-and-only.

[28] Liao D.S., Grossi F.V., El Mehdi D., Gerber M.R., Brown D.M., Heier J.S., Wykoff C.C., Singerman L.J., Abraham P., Grassmann F. (2020). Complement C3 Inhibitor Pegcetacoplan for Geographic Atrophy Secondary to Age-Related Macular Degeneration: A Randomized Phase 2 Trial. Ophthalmology.

[29] Nadeem A., Malik I.A., Shariq F., Afridi E.K., Taha M., Raufi N., Naveed A.K., Iqbal J., Habte A. (2023). Advancements in the treatment of geographic atrophy: Focus on pegcetacoplan in age-related macular degeneration. Ann. Med. Surg..

[30] Di Carlo E., Augustin A.J. (2021). Prevention of the Onset of Age-Related Macular Degeneration. J. Clin. Med..

[31] Xu X., Ritz B., Coleman A., Liew Z., Deapen D., Lee E., Bernstein L., Pinder R., Marshall S., Heck J.E. (2020). Hypertension, antihypertensive medications use and risk of age-related macular degeneration in California Teachers Cohort. J. Hum. Hypertens..

[32] Lin J.B., Halawa O.A., Husain D., Miller J.W., Vavvas D.G. (2022). Dyslipidemia in age-related macular degeneration. Eye.

[33] Hwang S., Kang S.W., Kim S.J., Lee K.N., Han K., Lim D.H. (2023). Diabetes-Related Risk Factors for Exudative Age-Related Macular Degeneration: A Nationwide Cohort Study of a Diabetic Population. Investig. Ophthalmol. Vis. Sci..

[34] Vujosevic S., Alovisi C., Chakravarthy U. (2023). Epidemiology of geographic atrophy and its precursor features of intermediate age-related macular degeneration. Acta Ophthalmol..

[35] Ruan Y., Jiang S., Gericke A. (2021). Age-Related Macular Degeneration: Role of Oxidative Stress and Blood Vessels. Int. J. Mol. Sci..

[36] Hanus J., Anderson C., Wang S. (2015). RPE necroptosis in response to oxidative stress and in AMD. Ageing Res. Rev..

[37] Landeck L., Asadullah K., Amasuno A., Pau-Charles I., Mrowietz U. (2018). Dimethyl fumarate (DMF) vs. monoethyl fumarate (MEF) salts for the treatment of plaque psoriasis: A review of clinical data. Arch. Dermatol. Res..

[38] Linker R.A., Lee D.H., Ryan S., van Dam A.M., Conrad R., Bista P., Zeng W., Hronowsky X., Buko A., Chollate S. (2011). Fumaric acid esters exert neuroprotective effects in neuroinflammation via activation of the Nrf2 antioxidant pathway. Brain.

[39] Manai F., Amadio M. (2022). Dimethyl Fumarate Triggers the Antioxidant Defense System in Human Retinal Endothelial Cells through Nrf2 Activation. Antioxidants.

[40] Rosito M., Testi C., Parisi G., Cortese B., Baiocco P., Di Angelantonio S. (2020). Exploring the Use of Dimethyl Fumarate as Microglia Modulator for Neurodegenerative Diseases Treatment. Antioxidants.

[41] Brennan M.S., Matos M.F., Li B., Hronowski X., Gao B., Juhasz P., Rhodes K.J., Scannevin R.H. (2015). Dimethyl fumarate and monoethyl fumarate exhibit differential effects on KEAP1, NRF2 activation, and glutathione depletion in vitro. PLoS ONE.

[42] Feeney-Burns L., Hilderbrand E.S., Eldridge S. (1984). Aging human RPE: Morphometric analysis of macular, equatorial, and peripheral cells. Investig. Ophthalmol. Vis. Sci..

[43] Terman A., Brunk U.T. (2004). Lipofuscin. Int. J. Biochem. Cell Biol..

[44] Sparrow J.R., Parish C.A., Hashimoto M., Nakanishi K. (1999). A2E, a lipofuscin fluorophore, in human retinal pigmented epithelial cells in culture. Investig. Ophthalmol. Vis. Sci..

[45] Westlund B.S., Cai B., Zhou J., Sparrow J.R. (2009). Involvement of c-Abl, p53 and the MAP kinase JNK in the cell death program initiated in A2E-laden ARPE-19 cells by exposure to blue light. Apoptosis.

[46] Wang J., Iacovelli J., Spencer C., Saint-Geniez M. (2014). Direct effect of sodium iodate on neurosensory retina. Investig. Ophthalmol. Vis. Sci..

[47] Koster C., van den Hurk K.T., Ten Brink J.B., Lewallen C.F., Stanzel B.V., Bharti K., Bergen A.A. (2022). Sodium-Iodate Injection Can Replicate Retinal Degenerative Disease Stages in Pigmented Mice and Rats: Non-Invasive Follow-Up Using OCT and ERG. Int. J. Mol. Sci..

[48] Chowers G., Cohen M., Marks-Ohana D., Stika S., Eijzenberg A., Banin E., Obolensky A. (2017). Course of Sodium Iodate-Induced Retinal Degeneration in Albino and Pigmented Mice. Investig. Ophthalmol. Vis. Sci..

[49] Bhutto I.A., Ogura S., Baldeosingh R., McLeod D.S., Lutty G.A., Edwards M.M. (2018). An Acute Injury Model for the Phenotypic Characteristics of Geographic Atrophy. Investig. Ophthalmol. Vis. Sci..

[50] Wu S., Zheng F., Sui A., Wu D., Chen Z. (2024). Sodium-iodate injection can replicate retinal and choroid degeneration in pigmented mice: Using multimodal imaging and label-free quantitative proteomics analysis. Exp. Eye Res..

[51] Waldstein S.M., Vogl W.D., Bogunovic H., Sadeghipour A., Riedl S., Schmidt-Erfurth U. (2020). Characterization of Drusen and Hyperreflective Foci as Biomarkers for Disease Progression in Age-Related Macular Degeneration Using Artificial Intelligence in Optical Coherence Tomography. JAMA Ophthalmol..

[52] Geathers J.S., Grillo S.L., Karakoleva E., Campbell G.P., Du Y., Chen H., Barber A.J., Zhao Y., Sundstrom J.M. (2024). Sodium Iodate: Rapid and Clinically Relevant Model of AMD. Front. Biosci. (Landmark Ed.).

[53] Kiuchi K., Yoshizawa K., Shikata N., Moriguchi K., Tsubura A. (2002). Morphologic characteristics of retinal degeneration induced by sodium iodate in mice. Curr. Eye Res..

[54] Provis J.M., Penfold P.L., Cornish E.E., Sandercoe T.M., Madigan M.C. (2005). Anatomy and development of the macula: Specialisation and the vulnerability to macular degeneration. Clin. Exp. Optom..

[55] Handa J.T. (2012). How does the macula protect itself from oxidative stress?. Mol. Aspects Med..

[56] Nita M., Grzybowski A. (2016). The Role of the Reactive Oxygen Species and Oxidative Stress in the Pathomechanism of the Age-Related Ocular Diseases and Other Pathologies of the Anterior and Posterior Eye Segments in Adults. Oxid. Med. Cell Longev..

[57] Kim J., Lee Y.J., Won J.Y. (2021). Molecular Mechanisms of Retinal Pigment Epithelium Dysfunction in Age-Related Macular Degeneration. Int. J. Mol. Sci..

[58] Cho H.M., Jo Y.D., Choung S.Y. (2022). Protective Effects of Spirulina maxima against Blue Light-Induced Retinal Damages in A2E-Laden ARPE-19 Cells and Balb/c Mice. Nutrients.

[59] Seol A., Kim J.E., Jin Y.J., Song H.J., Roh Y.J., Kim T.R., Park E.S., Park K.H., Park S.H., Uddin M.S. (2024). Novel Therapeutic Effects of Euphorbia heterophylla L. Methanol Extracts in Macular Degeneration Caused by Blue Light in A2E-Laden ARPE-19 Cells and Retina of BALB/c Mice. Pharmaceuticals.

[60] Jomova K., Alomar S.Y., Alwasel S.H., Nepovimova E., Kuca K., Valko M. (2024). Several lines of antioxidant defense against oxidative stress: Antioxidant enzymes, nanomaterials with multiple enzyme-mimicking activities, and low-molecular-weight antioxidants. Arch. Toxicol..

[61] Li Y., Choi E.H., Han I. (2019). Regulation of Redox Homeostasis by Nonthermal Biocompatible Plasma Discharge in Stem Cell Differentiation. Oxid. Med. Cell Longev..

[62] Thorpe G.W., Fong C.S., Alic N., Higgins V.J., Dawes I.W. (2004). Cells have distinct mechanisms to maintain protection against different reactive oxygen species: Oxidative-stress-response genes. Proc. Natl. Acad. Sci. USA.

[63] Tretter V., Hochreiter B., Zach M.L., Krenn K., Klein K.U. (2021). Understanding Cellular Redox Homeostasis: A Challenge for Precision Medicine. Int. J. Mol. Sci..

[64] Kim J., Cho K., Choung S.Y. (2020). Protective effect of Prunella vulgaris var. L extract against blue light induced damages in ARPE-19 cells and mouse retina. Free Radic. Biol. Med..

[65] Moriguchi M., Nakamura S., Inoue Y., Nishinaka A., Nakamura M., Shimazawa M., Hara H. (2018). Irreversible Photoreceptors and RPE Cells Damage by Intravenous Sodium Iodate in Mice Is Related to Macrophage Accumulation. Investig. Ophthalmol. Vis. Sci..

[66] Liu Y., Li R., Xie J., Hu J., Huang X., Ren F., Li L. (2018). Protective Effect of Hydrogen on Sodium Iodate-Induced Age-Related Macular Degeneration in Mice. Front. Aging Neurosci..

[67] Subrahmanian S.M., Yerlikaya E.I., Sunilkumar S., Toro A.L., McCurry C.M., Grillo S.L., Barber A.J., Sundstrom J.M., Dennis M.D. (2024). Deletion of the stress response protein REDD1 prevents sodium iodate-induced RPE damage and photoreceptor loss. Geroscience.

[68] Upadhyay M., Bonilha V.L. (2024). Regulated cell death pathways in the sodium iodate model: Insights and implications for AMD. Exp. Eye Res..

[69] Chuang C.J., Wang M., Yeh J.H., Chen T.C., Tsou S.C., Lee Y.J., Chang Y.Y., Lin H.W. (2021). The Protective Effects of α-Mangostin Attenuate Sodium Iodate-Induced Cytotoxicity and Oxidative Injury via Mediating SIRT-3 Inactivation via the PI3K/AKT/PGC-1α Pathway. Antioxidants.

[70] Hussar P. (2022). Apoptosis regulators bcl-2 and caspase-3. Encyclopedia.

[71] Mashimo M., Onishi M., Uno A., Tanimichi A., Nobeyama A., Mori M., Yamada S., Negi S., Bu X., Kato J. (2021). The 89-kDa PARP1 cleavage fragment serves as a cytoplasmic PAR carrier to induce AIF-mediated apoptosis. J. Biol. Chem..

[72] Parish C.A., Hashimoto M., Nakanishi K., Dillon J., Sparrow J. (1998). Isolation and one-step preparation of A2E and iso-A2E, fluorophores from human retinal pigment epithelium. Proc. Natl. Acad. Sci. USA.

