# Supplementary material for: Retinal Protective Effect of Mono-Ethyl Fumarate in Experimental Age-Related Macular Degeneration via Anti-Oxidative and Anti-Apoptotic Alterations
Source: Int J Mol Sci. 2025 Feb 7;26(4):1413. doi: 10.3390/ijms26041413 (PMC11855399; doi:10.3390/ijms26041413)
Supplement: Supplementary file 1 [file ijms-26-01413-s001.zip › ijms-3373273-supplementary.pdf]

Supplementary Material

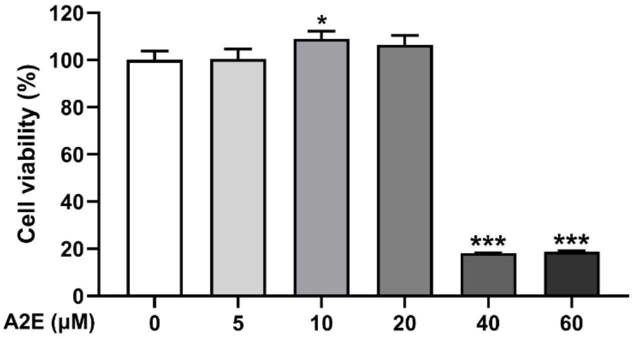

Figure S1. Cytotoxicity of A2E treatment on ARPE-19 cells. ARPE-19 cells were treated with various concentrations of A2E (5–60 μM) and incubated for 24 h to measure cell viability (N=3). *, *** Indicate significant differences (p < 0.05, 0.001) from the untreated group.

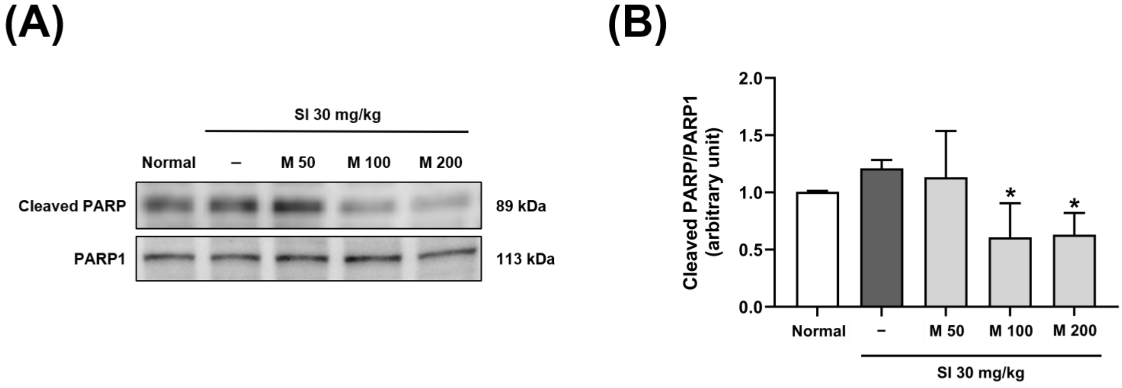

Figure S2. Analysis of PARP1 protein expression in the retina of SI-injected mice. Male C57BL/6J mice were orally administered with various doses of MEF (50–200 mg/kg) once daily for 4 weeks. A single round of intraperitoneal injection of SI at a dose of 30 mg/kg was performed at the beginning of the second week. (**A**) Representative images of western blot bands targeting cleaved PARP and PARP1 proteins from three independent experimental mice (N=3). (**B**) The expression level of cleaved PARP protein was normalized to PARP1 protein from experimental mice. All data are expressed as the mean ± SD. * Indicate a significant difference (p < 0.05) from the SI-injected group. SI, Sodium iodate; M, mono-ethyl fumarate.
